# Retention in Telehealth-Delivered Buprenorphine Treatment for Opioid Use Disorder: Multistate Retrospective Cohort Study

**DOI:** 10.2196/100527

**Published:** 2026-08-14

**Authors:** Laura B Monico, Elizabeth A Bambury, Jennifer K Foreman, Melody Avila, Stephen A Martin

**Affiliations:** 1 Boulder Care, 111 SW Naito Parkway, Suite 200, Portland, OR, 97204, United States, 1 (503) 208-5061; 2 Department of Family Medicine and Community Health, University of Massachusetts Chan Medical School, Worcester, MA, United States

**Keywords:** buprenorphine, telehealth, retention in treatment, medications for opioid use disorder, MOUD, virtual care, opioid use disorder, OUD

## Abstract

**Background:**

Buprenorphine is an effective treatment for opioid use disorder and is associated with reduced mortality when retention is high; however, national studies show low retention. Evidence from outpatient settings, including telehealth, remains limited, despite population-level analyses suggesting improved retention with virtual care. Further research is needed to better understand retention and optimize care for people with opioid use disorder.

**Objective:**

This study aimed to describe patient retention in a telehealth-delivered buprenorphine program over 24 months, both overall and by treatment transition pathway.

**Methods:**

We conducted a retrospective cohort study of adults initiating opioid use disorder treatment in a multistate, telehealth-only addiction treatment program between May 1, 2019, and April 30, 2025. Before March 2020, participants completed an in-person visit before transitioning to telehealth. Participants completed an intake appointment and either continued on a stable dose of buprenorphine started prior to care entry or transitioned to buprenorphine treatment via 1 of 6 treatment pathways (high dose, standard low dose, standard, Quick Start, low dose, or other). Retention in care was defined by ongoing clinical engagement, specifically through a completed medical visit or a patient-initiated secure message, each of which was required to continue buprenorphine treatment. Retention was analyzed both overall and by patient characteristics and treatment pathway. Chi-square tests and absolute risk differences with 95% CIs were used for comparisons.

**Results:**

Among 22,064 patients, the mean age was 41.2 (SD 9.9) years, 48.6% (10,542/21,676) were female, 33.8% (7464/22,064) lived in rural areas, 78.5% (16,826/21,446) were enrolled in Medicaid, and 37.1% (8181/22,064) transitioned to buprenorphine. Retention declined from 86.8% (19,154/22,064) at month 1 to 71.0% (11,488/16,183) at month 6 and then declined gradually through 24 months. At month 6, retention was higher among patients with prior buprenorphine exposure (8613/12,230, 70.4% vs 586/1128, 52.0%; risk difference: 18.5 percentage points, 95% CI 15.5%‐21.5%). Retention varied by pathway, with high dose showing the highest early retention (2330/2870, 81.2% at month 1), while patients already receiving buprenorphine at intake had higher retention than all other transition pathways.

**Conclusions:**

In this large, multistate study of telehealth-only buprenorphine treatment, retention was comparable to or markedly higher than national benchmarks. These findings underscore the potential of low-threshold, harm-reduction telehealth models to support long-term retention in opioid use disorder care during a period in which illicitly manufactured fentanyl predominates in the drug supply.

## Introduction

First declared in 2017, a Public Health Emergency for the opioid crisis remains in effect [1]. Although US opioid overdose mortality has slowed, deaths remain at December 2019 levels and continue to be the leading cause of death among individuals aged 18 to 44 years [2-4]. Aggregate improvements mask worsening outcomes among Black and Native American populations [5]. Meanwhile, the number of people affected continues to rise: an estimated 7.5% of US adults reported past-year use of illicitly manufactured fentanyl (IMF) in a recent national survey, and an estimated 9.4 million Americans require treatment for opioid use disorder (OUD) [6,7].

Methadone and buprenorphine remain the cornerstone of OUD care, each being associated with mortality reductions exceeding 50% when sustained long-term [8]. For buprenorphine, retention has declined for unclear reasons, potentially related to IMF exposure [9-11]. The 180-day retention benchmark—a quality standard—ranges from 22% to 30% in current national samples [9,12,13]. These low rates limit the benefit of treatment [14-16], and clinical trials have demonstrated minimal improvements in patient retention to date [17-19]. The timing of attrition is salient: early discontinuation (within 48 h) may reflect insurance or pharmacy barriers, or difficulty with initiation itself, such as buprenorphine-related precipitated withdrawal or an insufficient dose to suppress withdrawal symptoms, whereas attrition after several months may reflect changes in motivation or clinical status [20].

Despite most medication treatment occurring in ambulatory settings, outpatient-focused retention research remains scarce [21]. Protocols developed for emergency department or inpatient settings are often extrapolated to outpatient care, despite major differences. Inpatient-based reviews report successful transition to buprenorphine in up to 96% of low-dose transitions [22], and outpatient transition rates of 62% in single-arm observational studies and 82% in case reports have also been described [23]. By contrast, a recent retrospective outpatient analysis of low-dose transitions found only 22% retention with buprenorphine at 28 days [24]. These discrepancies highlight the limits of applying inpatient-derived and case-report evidence to outpatient practice and the need for high-quality data from routine ambulatory care [21,25]. Key questions in the IMF era—each of which requires stronger outpatient evidence—include buprenorphine’s effectiveness among people using IMF, the relative merits of transition methods, and demographic or clinical factors shaping initiation and continuation.

Newer modalities of outpatient care also require additional understanding. Telehealth-only OUD care was permitted by pandemic-era regulatory changes, which have since been extended. Population-level analyses of virtual care find improved retention and a lower risk of medically treated overdose compared with traditional care, but there are limited reports from individual settings [26-28].

Boulder Care, a multistate, telehealth-only clinic primarily serving patients with Medicaid, leveraged its experience providing care to more than 20,000 patients since the pandemic’s onset to examine retention, providing the scale and follow-up to inform outpatient retention in the IMF era [29]. This paper aims to evaluate whether 6-month and longer-term patient retention in a telehealth-delivered buprenorphine program exceeds current national benchmarks, whether retention differs across buprenorphine transition pathways, and whether specific patient characteristics are associated with higher retention rates than program averages. To our knowledge, this is the most comprehensive analysis of outpatient telehealth-only buprenorphine retention reported to date.

## Methods

### Study Design and Setting

This retrospective cohort study evaluated treatment retention among adults initiating OUD care through Boulder Care, a multistate, telehealth-only addiction treatment program, from May 2019 through April 2025. Care is grounded in harm-reduction and low-threshold access principles [29,30]. This study was reported in accordance with the STROBE (Strengthening the Reporting of Observational Studies in Epidemiology) guidelines for observational research (Checklist 1).

### Participants

Eligible participants were adults aged 18 years or older who enrolled in OUD treatment during the study period and completed a clinician-led intake visit. Enrollment required completion of this encounter; therefore, individuals who initiated but did not complete intake were not captured. Prior to March 2020, an in-person visit was required before patients transitioned to telehealth care. No additional exclusion criteria were applied. For each follow-up interval, denominators included only patients who had sufficient time in care to be eligible for retention assessment. All patients included in this analysis were receiving buprenorphine-related care at the time they were considered for retention metrics.

### Data Sources and Variables

Data were extracted from Boulder Care’s proprietary electronic health record, which uses structured fields completed at clinical encounters and internal validation (predefined selection categories, data-entry logic to reduce typographical errors, and chart review). Baseline variables included age, gender or sex, state of residence, rural versus urban residence, insurance type, prior buprenorphine exposure, stimulant use, and peer-support usage. The gender or sex field was determined using, in order of precedence, the gender value, if available; otherwise, legal gender; and, if neither is available, sex. Rural status was defined using the rural approximation crosswalk to the patient ZIP code from the Federal Office of Rural Health Policy. Peer support could occur at intake or during care. Stimulant use was documented by patient self-report at baseline.

### Exposure: Buprenorphine Transition Pathways

Among patients requiring a transition at intake, methods were grouped into 6 pathways: high dose, low dose, standard, standard low dose, Quick Start, or other (Table S1 in Multimedia Appendix 1). Pathway choice was determined through shared decision-making between the patient and prescriber, with patients selecting the approach most appropriate for their clinical and personal circumstances [31]. Systematic recording of the transition pathway field began in October 2022. Patients who enrolled before that date were issued a null value and classified as not requiring a transition by default. To confirm that the resulting early cohort did not distort pathway-stratified estimates, we conducted a sensitivity analysis among patients enrolled on or after October 2022 (Table S2 in Multimedia Appendix 1). Operational definitions of transition pathways followed the Boulder Care clinical resources [32-36]. Quick Start corresponded to a rapid naloxone-induced initiation (Table S1 in Multimedia Appendix 1 for detailed pathway descriptions) [37].

### Outcomes

The primary outcome was retention in care. A patient was considered retained in a given month if the clinical record documented an active clinical relationship, evidenced by either (1) a completed medical visit or (2) a patient-initiated secure message to the care team (Figure S1—Patient A in Multimedia Appendix 1). Each of these qualifying activities presupposes an ongoing therapeutic relationship and is a precondition for continued buprenorphine prescribing in this program, so either activity serves as an indicator that the patient remained engaged in care.

Eligibility for assessment was determined separately from retention. A patient entered the denominator for a given month only after enough time in the program had elapsed to reach it, allowing up to 105 days for a qualifying activity (completed visit or patient-initiated message) to occur so that clinically stable patients seen as infrequently as once per quarter were still considered retained in care. Among eligible patients, retention time accrued across the study period as qualifying activities continued. Analyses were conducted at the patient level. Individuals who enrolled in care more than once during the study period were represented once, with retained time measured from their index enrollment, and with qualifying activities in any episode of care contributing to that patient’s retained time. Patients with a gap of up to 105 days between activities were bridged and counted as retained in care. Therefore, months falling within a bridged interval were counted as retained, and a clinically stable patient seen every 60 to 90 days was considered retained during the months between visits (Figure S1—Patient B in Multimedia Appendix 1). For patients with a gap exceeding 105 days, retained time ended on the date of the last qualifying activity, and the days following that activity (including the 105-d allowance) were not counted as retained time (Figure S1—Patient C in Multimedia Appendix 1).

Given that retention in care is not directly observable and the addiction literature offers little consensus across studies, retention has been operationalized in numerous ways that yield materially different estimates [38]. The predominant approach within the field infers retention from continuous medication prescription coverage, typically through pharmacy-claims data, which capture only the availability of a dispensed medication rather than its consumption [39]. These approaches are particularly problematic in a low-barrier telehealth population, in which interruptions in coverage frequently reflect copayment and pharmacy access barriers, insurance changes, or medication rationing during periods of instability rather than a decision to disengage from care [40]. Patients who experience such interruptions often remain in contact and re-engage, and previous studies have measured retention as the cumulative sum of treatment days across reinitiation episodes, defining a new episode only after a sustained gap in attendance [41]. Therefore, measuring retention in care as sustained engagement rather than uninterrupted prescription coverage captures clinically meaningful outcomes for this population, which pharmacy claims–based data have been shown to systematically underestimate.

Secondary outcomes included retention stratified by transition pathway, with comparisons at each time point. Prespecified subgroup analyses examined 6-month retention by gender or sex, age, insurance type, rurality, prior buprenorphine exposure, stimulant use, peer-support engagement, and state. Parallel analyses at 1 month (early engagement) and 24 months (long-term retention) were conducted as secondary and exploratory outcomes, respectively.

### Statistical Analysis

Descriptive statistics characterized the cohort (means with SDs for continuous variables and counts with percentages for categorical variables). Retention was calculated as the number and percentage of eligible patients retained at each time point. Pathway comparisons used chi-square tests of independence (2-sided *α*=.05). Absolute risk differences (RDs) and 95% CIs were estimated using the Newcombe method for 2 independent proportions. Subgroup analyses at 6 months used the same approach, with RDs and 95% CIs reported for each category relative to a prespecified reference group; chi-square *P* values were provided for overall subgroup differences. No adjustments were made for multiple comparisons. Analyses were conducted using Stata version 18 (StataCorp LLC).

### Ethical Considerations

The WCG Institutional Review Board reviewed the protocol and determined that the analysis was exempt from oversight of research involving human participants.

## Results

### Study Cohort

During the study period, 22,064 patients initiated telehealth-based OUD treatment with Boulder Care (Table 1). The mean (SD) age was 41.2 (9.9) years, and 48.6% (10,542/21,676) were female patients. Most patients resided in Ohio (12,822/22,064, 58.1%), followed by Oregon (3665/22,064, 16.6%), Washington (2936/22,064, 13.3%), and North Carolina (2356/22,064, 10.7%); 1.3% (285/22,064) lived in other states. One-third (7464/22,064, 33.8%) lived in rural areas. Medicaid was the predominant insurance type (16,826/21,446, 78.5%), with 17.0% (3648/21,446) commercially insured and 4.5% (972/21,446) covered by Medicare. Lifetime prior buprenorphine exposure was reported by 91.9% (17,608/19,168) of patients. Co-occurring stimulant use was documented in 20.5% (4,520/22,064) of patients, and 51.2% (11,304/22,064) engaged in peer support.

At intake, 8181 patients (37.1%) required a buprenorphine transition, while 13,883 (62.9%) entered care already taking buprenorphine. Patients not requiring a transition were similar to the overall cohort but reported less stimulant use (2332/13,883, 16.8% vs 4520/22,064, 20.5%) and were slightly more likely to have commercial insurance (2610/13,495, 19.3% vs 3638/21,446, 17.0%). Stimulant use was more common in low-dose (128/345, 37.1%), Quick Start (153/450, 34.0%), standard low dose (797/2441, 32.7%), and other (45/147, 30.6%) groups compared with standard (357/1928, 18.5%). The low dose group also included more female patients (212/338, 62.7%). Prior buprenorphine exposure was common across all groups but lower in the standard low dose (1821/2220, 82.0%), Quick Start (338/411, 82.2%), and low dose (257/310, 82.9%) groups compared with the standard (1608/1883, 85.4%). Peer-support engagement ranged from 44.6% (154/345) to 59.3% (267/450), with the highest in Quick Start.

**Table 1.** Sample demographic and clinical characteristics both overall and by buprenorphine transition pathway.

Characteristics	Buprenorphine transition pathway							
	Overall (N=22,064)	No transition needed (n=13,883)	High dose (n=2870)	Standard low dose (n=2441)	Standard (n=1928)	Quick Start (n=450)	Low dose (n=345)	Other (n=147)
Age (y), mean (SD)	41.2 (9.9)	41.6 (9.9)	40.8 (9.8)	40.7 (9.9)	40.6 (9.9)	39.0 (8.2)	40.5 (9.4)	39.3 (9.6)
Gender/sex^a^, n (%)								
Female	10,542 (48.6)	6520 (47.9)	1379 (48.6)	1211 (50.2)	932 (49.5)	221 (50.2)	212 (62.7)	67 (45.9)
State of residence, n (%)								
Oregon	3665 (16.6)	2083 (15.0)	569 (19.8)	537 (22.0)	210 (10.9)	92 (20.4)	125 (36.2)	49 (33.3)
Washington	2936 (13.3)	1628 (11.7)	375 (13.1)	573 (23.5)	161 (8.4)	68 (15.1)	108 (31.3)	23 (15.6)
Ohio	12,822 (58.1)	8463 (61.0)	1662 (57.9)	1124 (46.0)	1160 (60.2)	253 (56.2)	90 (26.1)	70 (47.6)
North Carolina	2356 (10.7)	1525 (11.0)	220 (7.7)	177 (7.3)	385 (20.0)	29 (6.4)	16 (4.6)	4 (2.7)
Other^b^	285 (1.3)	184 (1.3)	44 (1.5)	30 (1.2)	12 (0.6)	8 (1.8)	6 (1.7)	1 (0.7)
Insurance type^a^, n (%)								
Medicaid	16,826 (78.5)	10,226 (75.8)	2335 (83.8)	1952 (82.3)	1519 (81.0)	386 (88.1)	293 (87.5)	115 (79.3)
Commercial	3648 (17.0)	2610 (19.3)	353 (12.7)	307 (12.9)	277 (14.8)	46 (10.5)	30 (9.0)	25 (17.2)
Medicare	972 (4.5)	659 (4.9)	99 (3.6)	112 (4.7)	79 (4.2)	6 (1.4)	12 (3.6)	5 (3.4)
Rural, n (%)	7464 (33.8)	4873 (35.1)	977 (34.0)	732 (30.0)	634 (32.9)	111 (24.7)	91 (26.4)	46 (31.3)
Past buprenorphine use^a^, n (%)	17,608 (91.9)	11,210 (97.0)	2261 (85.2)	1821 (82.0)	1608 (85.4)	338 (82.2)	257 (82.9)	113 (85.0)
Stimulant use, n (%)	4520 (20.5)	2332 (16.8)	708 (24.7)	797 (32.7)	357 (18.5)	153 (34.0)	128 (37.1)	45 (30.6)
Used peer support, n (%)	11,304 (51.2)	7128 (51.3)	1566 (54.6)	1194 (48.9)	919 (47.7)	267 (59.3)	154 (44.6)	76 (51.7)
Transition at intake, n (%)	8181 (37.1)	0 (0)	2870 (100)	2441 (100)	1928 (100)	450 (100)	345 (100)	147 (100)

a Sample sizes for gender/sex, insurance status, and prior buprenorphine experience are smaller than the total sample size because clinical data collection for these variables began after the start of the study period.

b Other states of residence included Alabama, Alaska, Arizona, California, Colorado, Florida, Idaho, Indiana, Kentucky, Michigan, Mississippi, Montana, Pennsylvania, South Carolina, Texas, Utah, Virginia, and West Virginia.

### Retention Over Time

**Figure 1.**
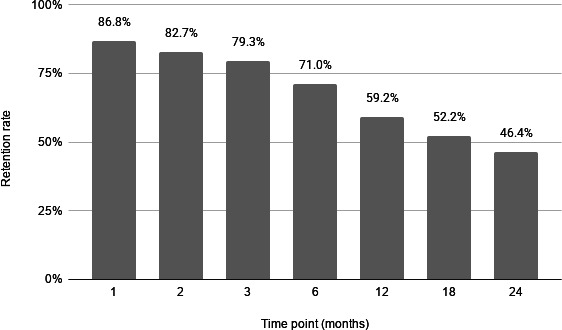
Retention from 1 to 24 months in telehealth-based opioid use disorder treatment.

Retention declined over time but remained substantial across 24 months (Figure 1; Table S3 in Multimedia Appendix 1). At 1 month, 19,154 out of 22,064 (86.8%) patients were retained. By 2 months, 17,291 out of 20,901 (82.7%) patients remained, and by 3 months, 15,541 out of 19,607 (79.3%) patients were engaged in care. At 6 months—a National Committee for Quality Assurance benchmark [9] —11,485 out of 16,184 (71.0%) patients were retained. By 12 months, 7038 out of 11,897 (59.2%) patients remained; by 18 months, retention was 4563 out of 8740 (52.2%) patients; and at 24 months, 2691 out of 5805 (46.4%) patients continued in care. Attrition was steeper in the first 6 months and then more gradual through 24 months.

### Retention by Demographic and Clinical Subgroups

At 6 months, retention differed across subgroups (Table 2). Rates were lowest among adults aged 18 to 24 years (120/269; 44.6%, 95% CI 38.8%‐50.6%) and highest among those aged 55 years or older (1356/1780; 76.2%, 95% CI 74.1%‐78.1%). Medicaid-insured patients had lower retention than commercially insured patients (8472/12,375, 68.5% vs 2162/2627, 82.3%; RD 13.8 percentage points, 95% CI 12.1%‐15.5%). Patients with prior buprenorphine exposure had higher retention (8613/12,230, 70.4% vs 586/1128, 52.0%; RD –18.5 percentage points, 95% CI –21.5% to –15.5%), while those reporting stimulant use had lower retention (2368/3665, 64.6% vs 9117/12,519, 72.8%; RD –8.2 percentage points, 95% CI –10.0% to –6.5%). Peer-support engagement was associated with markedly higher retention (7644/9099, 84.0% vs 3841/7085, 54.2%; RD –29.8 percentage points, 95% CI, –31.2% to –28.4%). Retention differed substantially by state and modestly by rural and urban residence.

Patterns at 1 and 24 months were consistent with these findings. At 1 month, overall retention was high (19,154/22,064, >86.8%), with stronger early engagement among patients with prior buprenorphine exposure and those using peer support and lower rates among younger adults and patients reporting stimulant use (Table S4 in Multimedia Appendix 1). By 24 months, overall retention declined to 46.4% (2691/5805), with persistent subgroup differences: older adults and patients engaged in peer support remained most likely to continue treatment, while young adults and those with stimulant use had the lowest long-term retention (Table S5 in Multimedia Appendix 1).

**Table 2.** Retention in care and risk differences at 6 months by demographic and clinical characteristics.

Characteristics	Eligible, n^a^	Retained^b^, n	Retained, % (95% CI)	RD^c^, % (95% CI)^d^	*P* value^e^
Age group (y)					<.001
25‐34	3864	2485	64.3 (62.8 to 65.8)	Reference	
18‐24	269	120	44.6 (38.8 to 50.6)	−19.7 (−25.7 to −13.5)	
35‐44	6857	4956	72.3 (71.2 to 73.3)	8.0 (6.1 to 9.8)	
45‐54	3414	2568	75.2 (73.7 to 76.6)	10.9 (8.8 to 13.0)	
≥55	1780	1356	76.2 (74.1 to 78.1)	11.9 (9.3 to 14.3)	
Gender/sex^f^					<.001
Female	7792	5723	73.4 (72.5 to 74.4)	Reference	
Male	8392	5762	68.7 (67.7 to 69.6)	−4.8 (−6.2 to −3.4)	
State					<.001
Ohio	9097	7030	77.3 (76.4 to 78.1)	Reference	
Oregon	3236	1889	58.4 (56.7 to 60.1)	−18.9 (−20.8 to −17.0)	
North Carolina	1151	899	78.1 (75.6 to 80.4)	0.8 (−1.8 to 3.3)	
Washington	2444	1480	60.6 (58.6 to 62.5)	−16.7 (−18.9 to −14.6)	
Other^g^	256	187	73.0 (67.3 to 78.1)	−4.2 (−10.0 to 0.9)	
Insurance type^f^					<.001
Medicaid	12,375	8472	68.5 (67.6 to 69.3)	Reference	
Commercial	2627	2162	82.3 (80.8 to 83.7)	13.8 (12.1 to 15.5)	
Medicare	709	568	80.1 (77.0 to 82.9)	11.7 (8.5 to 14.5)	
Rurality					<.001
Urban	10,681	7447	69.7 (68.8 to 70.6)	Reference	
Rural	5503	4038	73.4 (72.2 to 74.5)	3.7 (2.2 to 5.1)	
Past buprenorphine use^f^					<.001
Yes	12,230	8613	70.4 (69.6 to 71.2)	Reference	
No	1128	586	52.0 (49.0 to 54.9)	−18.5 (−21.5 to −15.5)	
Co-occurring stimulant use					<.001
No	12,519	9117	72.8 (72.0 to 73.6)	[Reference]	
Yes	3665	2368	64.6 (63.0 to 66.1)	−8.2 (−10.0 to −6.5)	
Used peer support					<.001
Yes	9099	7644	84.0 (83.2 to 84.7)	Reference	
No	7085	3841	54.2 (53.1 to 55.4)	−29.8 (−31.2 to −28.4)	
Transition at intake					<.001
No	10,085	7905	78.4 (77.6 to 79.2)	Reference	
Yes	6099	3580	58.7 (57.5 to 59.9)	−19.7 (−21.2 to −18.2)	

a Eligible at 6 months includes only patients with sufficient time in program to be assessed, allowing up to 105 days for a qualifying activity to account for stable patients seen quarterly. Total eligible patients (n=16,183).

b Retention is defined in the “Methods” section.

c RD: risk difference.

d RDs reflect the absolute percentage-point difference in retention compared with the reference category; 95% CIs were calculated using the Newcombe method.

e Chi-square *P* values indicate overall differences across subgroup categories; no adjustments were made for multiple comparisons.

f Sample sizes for gender/sex, insurance status, and prior buprenorphine experience are smaller than the total sample size because clinical data collection for these variables began after the start of the study period.

g Other states included Alabama, Alaska, Arizona, California, Colorado, Florida, Idaho, Indiana, Kentucky, Michigan, Mississippi, Montana, Pennsylvania, South Carolina, Texas, Utah, Virginia, and West Virginia.

### Retention by Transition Pathway

**Figure 2.**
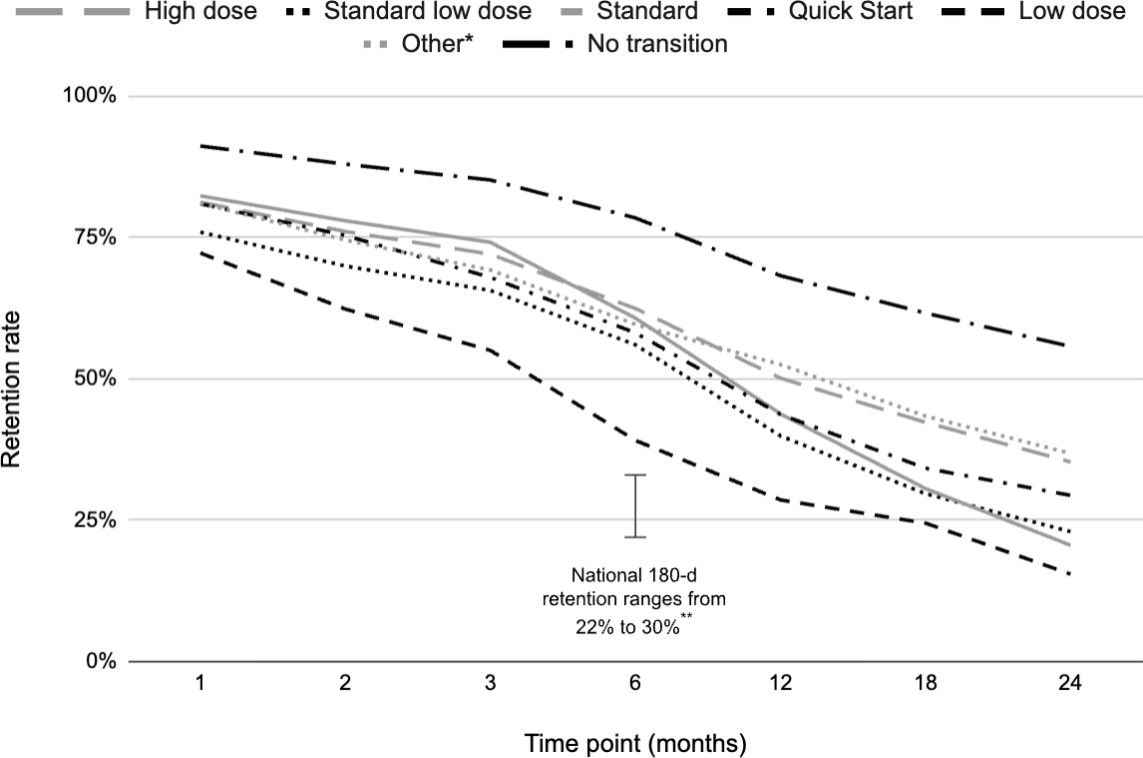
Retention over time by buprenorphine transition pathway in telehealth treatment for opioid use disorder. *“Other” includes patients who did not fit into established categories at the time and were classified based on clinical determination. **National rates are sourced from Pharmacotherapy for Opioid Use Disorder (POD). National Committee for Quality Assurance (NCQA) [9]; Chua et al [12]; and Olfson et al [13].

Among the 8181 patients requiring a buprenorphine transition at intake, outcomes differed by pathway (Figure 2; Table S3 in Multimedia Appendix 1). High dose yielded the highest retention, with 81.2% (2330/2870) retained at 1 month, 62.4% (1625/2604) at 6 months, and 35.3% (347/984) at 24 months. Standard and Quick Start showed intermediate outcomes, with 6-month retention rates of 60.7% (424/699) and 58.1% (211/363), respectively. Standard low dose had slightly lower retention (1131/2021, 56.0% at 6 mo). Low dose yielded the lowest rates, with just 39.1% (108/276) retained at 6 months and 15.5% (18/116) at 24 months. Patients classified as “other” showed relatively favorable retention (81/136, 59.6% at 6 mo and 25/68, 36.8% at 24 mo). By contrast, the 13,883 patients not requiring a transition achieved consistently higher retention, with 78.4% (7905/10,085) retained at 6 months and 55.7% (2094/3760) at 24 months.

Retention outcomes varied significantly between pathways (Table 3). Compared with low dose, high dose had consistently higher retention, with RDs peaking at 23.3% (95% CI 15.7%-30.9%) at 6 months and remaining elevated through 24 months (RD 19.7%, 95% CI 10.2%-29.3%). Compared with standard low dose, high dose showed smaller but significant differences, including 6.4% (95% CI 2.4%-10.5%) at 6 months and 12.3% (95% CI 6.1%-18.5%) at 24 months. Differences between high dose and Quick Start or Other were not significant at most time points. Standard also outperformed low dose, particularly at 3 months (RD 19.1%, 95% CI 11.3%-26.9%) and 6 months (RD 21.5%, 95% CI 12.2%-30.9%), with effects narrowing by 24 months (RD 5.1%, 95% CI –9.4% to 19.7%). Comparisons between standard and standard low dose showed modest early differences that diminished by 12 months. Quick Start and Other exhibited similar trajectories, while low dose remained consistently lowest in retention. A sensitivity analysis restricting pathway comparisons to patients enrolled on or after October 2022 due to changes in clinical data collection produced consistent rankings and RDs (Table S2 in Multimedia Appendix 1), indicating that any potential misclassification of transition pathway in this early cohort of patients would actually attenuate rather than inflate observed differences between transition and nontransition groups, and therefore demonstrating that the findings of this analysis are not affected by these data capture changes.

**Table 3.** Risk differences (RDs) in retention by buprenorphine transition pathway at treatment time points^a^.

Transition pathway	RD, % (95% CI)^b^ and retention time point^c^						
	Month 1	Month 2	Month 3	Month 6	Month 12	Month 18	Month 24
High dose vs low dose	9.0 (2.9 to 15.1)	13.7 (6.9 to 20.5)	17.0 (9.8 to 24.2)	23.3 (15.7 to 30.9)	21.5 (13.6 to 29.5)	17.8 (8.7 to 26.9)	19.7 (10.2 to 29.3)
High dose vs other^d^	0.2 (−7.5 to 8.0)	1.5 (−7.1 to 10.1)	2.7 (−6.4 to 11.9)	2.8 (−7.2 to 12.8)	−2.3 (−14.0 to 9.3)	−1.1 (−13.9 to 11.8)	−1.5 (−15.7 to 12.7)
High dose vs Quick Start	0.3 (−4.8 to 5.4)	0.7 (−5.0 to 6.3)	4.0 (−2.1 to 10.2)	4.3 (−2.6 to 11.2)	6.5 (−1.3 to 14.2)	8.1 (−0.8 to 17.1)	5.9 (−5.2 to 16.9)
High dose vs standard	−1.1 (−4.3 to 2.0)	−1.9 (−5.4 to 1.7)	−2.1 (−6.1 to 1.8)	1.7 (−3.7 to 7.2)	6.4 (−2.2 to 14.9)	11.8 (1.9 to 21.6)	14.6 (3.7 to 25.6)
High dose vs standard low dose	5.3 (2.1 to 8.4)	6.1 (2.7 to 9.6)	6.3 (2.7 to 10.0)	6.4 (2.4 to 10.5)	10.3 (5.8 to 14.8)	12.6 (7.4 to 17.7)	12.3 (6.1 to 18.5)
Low dose vs other	−8.8 (−19.8 to 2.2)	−12.2 (−24.4 to 0.1)	−14.2 (−27.2 to −1.3)	−20.4 (−34.3 to −6.6)	−23.8 (−39.2 to −8.4)	−18.8 (−35.9 to −1.8)	−21.2 (−39.0 to −3.5)
Quick Start vs low dose	8.7 (0.4 to 17.1)	13.0 (3.8 to 22.3)	13.0 (3.0 to 22.9)	19.0 (8.2 to 29.8)	15.1 (3.6 to 26.5)	9.6 (−3.5 to 22.8)	13.9 (−0.8 to 28.6)
Quick Start vs other	−0.1 (−10.0 to 9.9)	0.9 (−10.2 to 12.0)	−1.3 (−13.2 to 10.7)	−1.4 (−14.6 to 11.8)	−8.8 (−24.0 to 6.4)	−9.2 (−26.1 to 7.7)	−7.4 (−26.6 to 11.9)
Standard vs low dose	10.1 (3.7 to 16.6)	15.6 (8.4 to 22.8)	19.1 (11.3 to 26.9)	21.5 (12.2 to 30.9)	15.1 (2.8 to 27.4)	6.0 (−8.1 to 20.1)	5.1 (−9.4 to 19.7)
Standard vs other	1.4 (−6.7 to 9.4)	3.4 (−5.6 to 12.4)	4.9 (−4.9 to 14.7)	1.1 (−10.7 to 12.8)	−8.7 (−24.7 to 7.3)	−12.8 (−30.7 to 5.1)	−16.1 (−35.3 to 3.0)
Standard vs Quick Start	1.4 (−3.9 to 6.8)	2.5 (−3.5 to 8.6)	6.2 (−0.6 to 12.9)	2.5 (−6.1 to 11.2)	0.1 (−12.0 to 12.1)	−3.6 (−17.5 to 10.3)	−8.8 (−24.9 to 7.3)
Standard vs standard low dose	6.4 (3.0 to 9.8)	8.0 (4.2 to 11.8)	8.5 (4.2 to 12.7)	4.7 (−1.1 to 10.5)	3.9 (−4.9 to 12.7)	0.8 (−9.3 to 11.0)	−2.4 (−13.6 to 8.8)
Standard low dose vs low dose	3.7 (−2.7 to 10.1)	7.6 (0.5 to 14.6)	10.6 (3.2 to 18.1)	16.8 (8.9 to 24.7)	11.2 (3.0 to 19.5)	5.2 (−4.1 to 14.6)	7.5 (−2.3 to 17.3)
Standard low dose vs other	−5.0 (−13.1 to 3.0)	−4.6 (−13.5 to 4.3)	−3.6 (−13.0 to 5.8)	−3.6 (−13.9 to 6.7)	−12.6 (−24.6 to −0.7)	−13.6 (−26.8 to −0.5)	−13.8 (−28.1 to 0.6)
Standard low dose vs Quick Start	−5.0 (−10.3 to 0.3)	−5.5 (−11.4 to 0.4)	−2.3 (−8.7 to 4.1)	−2.2 (−9.4 to 5.0)	−3.8 (−11.9 to 4.2)	−4.4 (−13.6 to 4.8)	−6.4 (−17.7 to 4.9)

a The transition pathway was systematically recorded after October 2022; patients enrolling earlier without a recorded pathway are overrepresented in the no transition needed group. A sensitivity analysis restricted to enrollments after October 2022 is reported in Table S2 in Multimedia Appendix 1.

b RDs reflect the absolute percentage-point difference in retention between groups at each time point; 95% CIs were calculated using the Newcombe method for 2 independent proportions. Positive values indicate higher retention in the first-listed group; negative values indicate higher retention in the second-listed group.

c Retention in care is defined in the “Methods” section. Eligible patients at each month include those with sufficient time in the program to be assessed for that month.

d Other includes patients who did not fit into established categories at the time and were classified based on clinical determination.

## Discussion

### Principal Findings

Our findings provide robust evidence that in a telehealth-only outpatient setting, buprenorphine retention markedly exceeds national benchmarks during the current period of widespread fentanyl exposure in the drug supply. In 2024, national 6-month retention was 25.9% for patients with Medicaid and 29.9% for patients with commercial health maintenance organization coverage [9]. In our cohort, 6-month retention reached 71.0% (11,485/16,184), a finding consistent with buprenorphine’s continued role as a first-line treatment. Retention was uniformly high at 1 month and declined to 46.4% (2691/5805) at 24 months, with subgroup patterns at both time points mirroring those observed at 6 months.

More than 60% of new patients were already taking buprenorphine at intake, which they obtained prior to enrollment from sources outside the program. This prevalence is consistent with prior studies and may reflect a range of stabilization pathways [42]. Patients continuing on buprenorphine (“No Transition”) demonstrated the strongest early retention, while those initiating treatment faced greater discontinuation risk, consistent with evidence that reducing opioid use often requires multiple attempts [43]. These findings highlight the reality that many individuals enter treatment already engaged in some form of self-management and underscore the clinical importance of supporting continued stabilization rather than unnecessarily restarting transition processes.

When stratified by transition pathway, high-dose and standard approaches produced the highest 1-month retention (~82%), while low dose lagged (~72%). The long-standing concern that standard transitions may provoke precipitated withdrawal or other adverse experiences has limited their use in outpatient settings [44]. Yet our results suggest clinical support and adequate dosing may mitigate these risks [45]. The favorable retention associated with high dose, previously reported only in case studies, is especially notable, pointing to its promise as a scalable outpatient strategy [46]. Quick Start, selected by 2% (450/22,064) of patients, yielded retention comparable to that of standard, meriting further investigation [37,45,47]. By contrast, low dose transitions were associated with the poorest outcomes, consistent with their design for inpatient settings where full agonists can be administered [11,46]. Outpatient instructions to initiate buprenorphine while continuing fentanyl are both confusing and difficult to follow in practice and may contribute to dropout [48]. These findings reinforce the value of shared decision-making: when presented with multiple transition strategies, patients favored high dose and standard, and these choices aligned with better retention. Patients’ ability to select a pathway that matches their circumstances appears central to engagement and merits further emphasis in future implementation studies.

Subgroup analyses confirmed established predictors of attrition—male gender or sex, younger age, and stimulant co-use—consistent with prior literature [14]. In contrast, rurality was associated with higher retention, diverging from earlier work but plausibly reflecting telehealth’s ability to remove transportation and geographic barriers [49]. Although Medicaid-insured individuals had lower 6-month retention than those with commercial insurance (8472/12,375, 68.5% vs 2162/2627, 82.3%), both rates significantly exceeded national benchmarks [9]. Particularly striking was the 20-percentage-point variation in retention between states, which raises important hypotheses about how local regulatory environments, health system infrastructure, and drug supply conditions may shape treatment continuity. Prior analyses have identified clusters of elevated substance use mortality across US counties, underscoring the need for region-specific interventions [50]. Our findings suggest that policy and practice strategies must account for these local contexts if retention is to be improved equitably nationwide.

Peer support was among the strongest protective factors observed, conferring an absolute improvement of nearly 30% in 6-month retention. While uptake of peer support reflects a self-selected group of patients, the magnitude of the benefit underscores its potential as a cornerstone for treatment models [51]. Expanding access to peer-delivered services within telehealth frameworks may be one of the most feasible and scalable strategies for improving engagement and retention.

Beyond these specific findings, our results add to the growing evidence supporting telehealth as an effective platform for OUD care [52]. Across months 1 through 24, our retention consistently exceeded national averages in a large, predominantly Medicaid-insured, unselected cohort. Retention remained nearly 50% at 24 months, underscoring the potential of telehealth models to sustain engagement over the long term. Entry to care was facilitated via audio or web-based modalities, often with support from community partners, minimizing infrastructure barriers. Although ongoing care requires access to a smartphone, our experience supports other findings that telehealth can reduce, rather than exacerbate, inequities in treatment access [53]. At a time when efforts to expand buprenorphine prescribing have not translated into proportional increases in treatment availability, telehealth represents an opportunity to scale evidence-based care rapidly and equitably. These results from a large outpatient cohort have important implications for clinical practice and related policy. First, they reinforce buprenorphine’s ongoing effectiveness during this period when fentanyl dominates the opioid drug supply as demonstrated by sustained retention in care; together with methadone, it should be routinely offered as treatment. Second, they provide direct empirical support for fentanyl-era transitions to buprenorphine grounded in individualization, shared decision-making, multiple pathways, and thoughtful clinical care [54]. Third, they align with models of low-threshold care that make it “easier to start and continue buprenorphine,” with principles that include patient-centeredness, same-day treatment initiation, harm reduction, and welcoming patients back to care, should there be a disconnection [55]. Fourth, with respect to policy, telehealth-based care’s ability to reliably offer these needed qualities has only been possible in the United States since 2020. This requires permanent legal and regulatory support [26,56].

### Strengths and Limitations

This study has notable strengths, including its large sample of more than 20,000 patients, an exclusively outpatient telehealth context during the fentanyl era, and the integration of shared decision-making in selecting initiation pathways. Limitations include its retrospective design and intention-to-treat assignment, in which the pathway selected by patients may not always reflect actual dosing behavior. This cohort study of patients enrolled in telehealth-only OUD treatment was not designed as a comparison trial with a control group. Race and ethnicity were not collected during this period, constraining analysis of equity. Similarly, because this study leverages clinical data from a virtual OUD treatment clinic, comprehensive and systematic data related to other variables of interest (eg, opioid use type at intake, number of previous medications for opioid use disorder or OUD treatment episodes, length of previous medications for opioid use disorder or OUD treatment episodes) were not available. We did not systematically assess precipitated withdrawal, and injectable buprenorphine, potentially associated with higher retention, was not available [57]. Patients also experienced a single model of care, though the low-threshold, harm-reduction approach is aligned with national recommendations [58,59]. Finally, although our cohort spanned 4 states, heterogeneity across states suggests that broader replication is needed to establish generalizability.

### Conclusions

This large, multistate cohort study demonstrates that telehealth-only buprenorphine care in the fentanyl era can achieve retention rates substantially higher than previously reported, reinforcing its role as a scalable, patient-centered, and low-threshold model of care. The ability of patients to select among transition strategies appears central to retention, with high-dose and standard pathways frequently chosen and yielding the most favorable early outcomes [44,60].

With more than 20,000 predominantly Medicaid-insured patients and follow-up extending to 24 months, this study provides the most comprehensive outpatient telehealth data on buprenorphine retention to date. High-dose initiation, peer support, and patient choice were strongly associated with improved retention, while state-level variation underscored the influence of local context. These findings suggest that telehealth can expand access, reduce inequities, and support engagement over time. Sustained retention in this large, real-world cohort reinforces the conclusion that patient-centered, low-threshold models can support durable buprenorphine treatment in the fentanyl era.

## Supplementary material

10.2196/100527 Multimedia Appendix 1 Transition pathway definitions, additional results and sensitivity analyses, figure data, and illustrative example of primary outcome.

10.2196/100527 Checklist 1 STROBE checklist.

## Abbreviations

IMF: illicitly manufactured fentanyl
OUD: opioid use disorder
RD: risk difference
STROBE: Strengthening the Reporting of Observational Studies in Epidemiology
