# Supplementary material for: Retention in Telehealth-Delivered Buprenorphine Treatment for Opioid Use Disorder: Multistate Retrospective Cohort Study
Source: J Med Internet Res. 2026 Aug 14;28:e100527. doi: 10.2196/100527 (PMC13475782; doi:10.2196/100527)
Supplement: Supplementary file 1 [file jmir-v28-e100527-s001.docx]

**Supplement**

eTable 1. Transition pathway definitions	2

eTable 2. Sensitivity analysis for patients enrolled post-October 2022: retention in care at treatment timepoints by buprenorphine transition pathway	3

eTable 3. Retention in care at treatment timepoints by buprenorphine transition pathway	4

eTable 4. Retention in care and risk differences at 1 month by demographic and clinical characteristics	5

eTable 5. Retention in care and risk differences at 24 Months by demographic and clinical characteristics	7

eFigure 1. Determination of retention in care: three illustrative patient examples	9

eTable 1. Transition pathway definitions

| **Transition method** | **Initiating dose**  **buprenorphine**  **(dual or monoproduct)** | **Initial dosing interval** | **Dose titration** | **Timeframe** |
| --- | --- | --- | --- | --- |
| Standard dose | 8 mg sublingual once in mild-moderate withdrawal | Every 8 hours | Total 16-24 mg first 24 hours | ≤ 24 hours |
| Standard low dose | 1–2 mg sublingual once in early-mild withdrawal | Every 1–2 hours as symptoms persist | Total 16-24 mg first 24 hours | ≤ 24 hours |
| Low dose (Bernese Method / Microdosing ) | 0.5 mg sublingual once a day | Once or twice daily | Increase by 0.5-1 mg daily while decreasing opioid agonist use | 5–10 days (sometimes longer) |
| High dose (Macrodosing) | 16 mg sublingual once in moderate withdrawal | Every 30–60 minutes | Take 8 mg additional if needed within 1 hour of the first dose if still in significant withdrawal to maximum total dose 24mg the first 24 hours | ≤ 24 hours |
| Quick Start | 24 mg sublingual immediately before self-administration of Intranasal Naloxone (together with premedication beforehand and social support throughout) | One-time dose of 24 mg | Can take additional 8 mg dose within 24 hours as needed for withdrawal symptoms | < 1 hour |

eTable 2. Sensitivity analysis for patients enrolled post-October 2022: retention in care at treatment timepoints by buprenorphine transition pathway

|  |  | **Retention rate, %** | | | | | | |
| --- | --- | --- | --- | --- | --- | --- | --- | --- |
|  | **N (%)^a^** | **Retention time point (mo)^b^** | | | | | | |
| **Transition pathwayᶜ** |  | **1**  **(n=19451)** | **2**  **(n=18288)** | **3**  **(n=16994)** | **6**  **(n=13571)** | **12**  **(n=9284)** | **18**  **(n=6127)** | **24**  **(n=3192)** |
| High dose | 2707 (13.9) | 80.4 | 75.1 | 71.2 | 61.5 | 49.4 | 42.0 | 36.1 |
| Standard low dose | 2253 (11.6) | 74.2 | 67.9 | 63.8 | 54.0 | 37.9 | 26.8 | 21.8 |
| Standard dose | 1898 (9.8) | 82.1 | 77.5 | 73.7 | 59.8 | 41.8 | 28.1 | 17.9 |
| Quick Start | 413 (2.1) | 79.4 | 73.8 | 66.4 | 55.8 | 40.6 | 31.5 | 26.8 |
| Low dose | 312 (1.6) | 69.2 | 58.4 | 51.8 | 35.8 | 25.8 | 22.2 | 13.3 |
| Other | 135 (0.7) | 79.3 | 72.2 | 67.9 | 57.3 | 52.8 | 45.1 | 39.3 |
| No transition | 11733 (60.3) | 90.5 | 87.3 | 84.3 | 77.0 | 66.0 | 59.4 | 54.1 |
| Overall | 19,451 (100.0) | 85.8 | 81.5 | 77.9 | 68.8 | 56.0 | 48.4 | 41.9 |

^a^ Eligible at each retention month include only patients with sufficient time-in-program to be assessed for retention; denominators decline over time accordingly.

^b^ Retention in care is defined in the Methods of the main manuscript.

ᶜ Transition pathways represent buprenorphine initiation method used at intake.

eTable 3. Retention in care at treatment timepoints by buprenorphine transition pathway

|  |  | **Retention rate, %** | | | | | | |
| --- | --- | --- | --- | --- | --- | --- | --- | --- |
|  | **N (%)ᵃ** | **Retention time point (mo)ᵇ** | | | | | | |
| **Transition pathwayᶜ** |  | **1**  **(n=22,064)** | **2**  **(n=20,901)** | **3**  **(n=19,607)** | **6**  **(n=16,183)** | **12**  **(n=11,897)** | **18**  **(n=8,740)** | **24**  **(n=5,805)** |
| High dose | 2,870 (13.0) | 81.2 | 76.0 | 72.0 | 62.4 | 50.1 | 42.3 | 35.3 |
| Standard low dose | 2,441 (11.1) | 75.9 | 69.9 | 65.6 | 56.0 | 39.9 | 29.7 | 23.0 |
| Standard dose | 1,928 (8.7) | 82.3 | 77.9 | 74.1 | 60.7 | 43.8 | 30.6 | 20.6 |
| Quick Start | 450 (2.0) | 80.9 | 75.3 | 67.9 | 58.1 | 43.7 | 34.2 | 29.4 |
| Low dose | 345 (1.6) | 72.2 | 62.3 | 55.0 | 39.1 | 28.6 | 24.5 | 15.5 |
| Other | 147 (0.7) | 81.0 | 74.5 | 69.2 | 59.6 | 52.5 | 43.4 | 36.8 |
| No transition | 13,883 (62.9) | 91.1 | 87.9 | 85.1 | 78.4 | 68.2 | 61.6 | 55.7 |
| Overall | 22,064 (100.0) | 86.8 | 82.7 | 79.3 | 71.0 | 59.2 | 52.2 | 46.4 |

^a^ Eligible at each retention month include only patients with sufficient time-in-program to be assessed for retention; denominators decline over time accordingly.

^b^ Retention in care is defined in the Methods of the main manuscript.

ᶜ Transition pathways represent buprenorphine initiation method used at intake.

eTable 4. Retention in care and risk differences at 1 month by demographic and clinical characteristics

| **Characteristic** | **Eligible, Nᵃ** | **Retained, nᵇ** | **Retained, % (95%CI)** | **RD, % (95% CI)ᶜ** | ***P* valueᵈ** |
| --- | --- | --- | --- | --- | --- |
| Age group |  |  |  |  |  |
| 25–34 | 5339 | 4505 | 84.4% (83.4–85.3%) | [Reference] | <.001 |
| 18–24 | 416 | 316 | 76.0% (71.6–79.8%) | -8.4 (-12.9 to -4.4) |  |
| 35–44 | 9357 | 8188 | 87.5% (86.8–88.2%) | 3.1 (2.0 to 4.3) |  |
| 45–54 | 4631 | 4103 | 88.6% (87.7–89.5%) | 4.2 (2.9 to 5.6) |  |
| ≥55 | 2321 | 2042 | 88.0% (86.6–89.2%) | 3.6 (1.9 to 5.2) |  |
| Gender/Sex |  |  |  |  |  |
| Female | 10542 | 9240 | 87.6% (87.0–88.3%) | [Reference] | <.001 |
| Male | 11522 | 9914 | 86.0% (85.4–86.7%) | -1.6 (-2.5 to -0.7) |  |
| State |  |  |  |  |  |
| Ohio | 12822 | 11557 | 90.1% (89.6–90.6%) | [Reference] | <.001 |
| North Carolina | 2356 | 2140 | 90.8% (89.6–91.9%) | 0.7 (-0.6 to 1.9) |  |
| Washington | 2936 | 2321 | 79.1% (77.5–80.5%) | -11.1 (-12.7 to -9.6) |  |
| Oregon | 3665 | 2887 | 78.8% (77.4–80.1%) | -11.4 (-12.8 to -10.0) |  |
| Other^e^ | 285 | 249 | 87.4% (83.0–90.7%) | -2.8 (-7.2 to 0.6) |  |
| Insurance type |  |  |  |  |  |
| Medicaid | 16826 | 14383 | 85.5% (84.9–86.0%) | [Reference] | <.001 |
| Commercial | 3648 | 3367 | 92.3% (91.4–93.1%) | 6.8 (5.8 to 7.8) |  |
| Medicare | 972 | 868 | 89.3% (87.2–91.1%) | 3.8 (1.7 to 5.7) |  |
| Rurality |  |  |  |  |  |
| Urban | 14600 | 12592 | 86.2% (85.7–86.8%) | [Reference] | <.001 |
| Rural | 7464 | 6562 | 87.9% (87.2–88.6%) | 1.7 (0.7 to 2.6) |  |
| Past buprenorphine |  |  |  |  |  |
| Yes | 17608 | 15281 | 86.8% (86.3–87.3%) | [Reference] | <.001 |
| No | 1560 | 1166 | 74.7% (72.5–76.8%) | -12.0 (-14.3 to -9.9) |  |
| Co-occurring stimulant |  |  |  |  |  |
| No | 17544 | 15440 | 88.0% (87.5–88.5%) | [Reference] | <.001 |
| Yes | 4520 | 3714 | 82.2% (81.0–83.3%) | -5.8 (-7.1 to -4.6) |  |
| Utilized peer support |  |  |  |  |  |
| Yes | 11304 | 10788 | 95.4% (95.0–95.8%) | [Reference] | <.001 |
| No | 10760 | 8366 | 77.8% (77.0–78.5%) | -17.7 (-18.6 to -16.8) |  |
| Transition at intake |  |  |  |  |  |
| No | 13883 | 12652 | 91.1% (90.6–91.6%) | [Reference] | <.001 |
| Yes | 8181 | 6502 | 79.5% (78.6–80.3%) | -11.7 (-12.7 to -10.7) |  |

Abbreviations: RD, Risk Difference

ᵃ Eligible at 1 month includes only patients with sufficient time in program to be assessed, allowing up to 105 days for a qualifying activity to account for stable patients seen quarterly. Total eligible N = 22,064.

ᵇ Retention in care is defined in the Methods of the main manuscript.

ᶜ Risk differences reflect the absolute percentage-point difference in retention compared with the reference category; 95% confidence intervals were calculated using the Newcombe method.

ᵈ Chi-square p values indicate overall differences across subgroup categories; no adjustments were made for multiple comparisons.

^e^ Other states include: Alabama, Alaska, Arizona, California, Colorado, Florida, Idaho, Indiana, Kentucky, Michigan, Mississippi, Montana, Pennsylvania, South Carolina, Texas, Utah, Virginia, and West Virginia.

eTable 5. Retention in care and risk differences at 24 Months by demographic and clinical characteristics

| **Characteristic** | **Eligible, Nᵃ** | **Retained, nᵇ** | **Retained, % (95%CI)** | **RD, % (95% CI)ᶜ** | ***P* valueᵈ** |
| --- | --- | --- | --- | --- | --- |
| Age group |  |  |  |  |  |
| 25–34 | 1362 | 508 | 37.3% (34.8–39.9%) | [Reference] | <.001 |
| 18–24 | 79 | 15 | 19.0% (11.9–29.0%) | -18.3 (-25.9 to -8.0) |  |
| 35–44 | 2439 | 1121 | 46.0% (44.0–47.9%) | 8.7 (5.4 to 11.9) |  |
| 45–54 | 1263 | 660 | 52.3% (49.5–55.0%) | 15.0 (11.2 to 18.7) |  |
| ≥55 | 662 | 387 | 58.5% (54.7–62.2%) | 21.2 (16.6 to 25.6) |  |
| Gender/Sex |  |  |  |  |  |
| Female | 2853 | 1484 | 52.0% (50.2–53.8%) | [Reference] | <.001 |
| Male | 2952 | 1207 | 40.9% (39.1–42.7%) | -11.1 (-13.7 to -8.6) |  |
| State |  |  |  |  |  |
| Ohio | 2537 | 1365 | 53.8% (51.9–55.7%) | [Reference] | <.001 |
| North Carolina | 238 | 136 | 57.1% (50.8–63.3%) | 3.3 (-3.3 to 9.8) |  |
| Oregon | 1693 | 644 | 38.0% (35.8–40.4%) | -15.8 (-18.8 to -12.7) |  |
| Washington | 1197 | 477 | 39.8% (37.1–42.7%) | -14.0 (-17.3 to -10.5) |  |
| Other^e^ | 140 | 69 | 49.3% (41.1–57.5%) | -4.5 (-12.9 to 3.9) |  |
| Insurance type |  |  |  |  |  |
| Medicaid | 4394 | 1844 | 42.0% (40.5–43.4%) | [Reference] | <.001 |
| Commercial | 951 | 620 | 65.2% (62.1–68.2%) | 23.2 (19.8 to 26.5) |  |
| Medicare | 281 | 168 | 59.8% (54.0–65.4%) | 17.8 (11.8 to 23.6) |  |
| Rurality |  |  |  |  |  |
| Urban | 3954 | 1804 | 45.6% (44.1–47.2%) | [Reference] | 0.11 |
| Rural | 1851 | 887 | 47.9% (45.7–50.2%) | 2.3 (-0.5 to 5.0) |  |
| Past buprenorphine |  |  |  |  |  |
| Yes | 2851 | 1252 | 43.9% (42.1–45.7%) | [Reference] | <.001 |
| No | 290 | 68 | 23.4% (18.9–28.7%) | -20.5 (-25.3 to -15.0) |  |
| Co-occurring stimulant |  |  |  |  |  |
| No | 4588 | 2222 | 48.4% (47.0–49.9%) | [Reference] | <.001 |
| Yes | 1217 | 469 | 38.5% (35.8–41.3%) | -9.9 (-13.0 to -6.8) |  |
| Utilized peer support |  |  |  |  |  |
| Yes | 3681 | 2144 | 58.2% (56.6–59.8%) | [Reference] | <.001 |
| No | 2124 | 547 | 25.8% (23.9–27.7%) | -32.5 (-34.9 to -30.0) |  |
| Transition at intake |  |  |  |  |  |
| No | 3760 | 2094 | 55.7% (54.1–57.3%) | [Reference] | <.001 |
| Yes | 2045 | 597 | 29.2% (27.3–31.2%) | -26.5 (-29.0 to -23.9) |  |

Abbreviations: RD, Risk Difference

ᵃ Eligible at 24 months includes only patients with sufficient time in program to be assessed, allowing up to 105 days for a qualifying activity to account for stable patients seen quarterly. Total eligible N = 5,805.

ᵇ Retention in care is defined in the Methods of the main manuscript.

ᶜ Risk differences reflect the absolute percentage-point difference in retention compared with the reference category; 95% confidence intervals were calculated using the Newcombe method.

ᵈ Chi-square p values indicate overall differences across subgroup categories; no adjustments were made for multiple comparisons.

^e^ Other states include: Alabama, Alaska, Arizona, California, Colorado, Florida, Idaho, Indiana, Kentucky, Michigan, Mississippi, Montana, Pennsylvania, South Carolina, Texas, Utah, Virginia, and West Virginia.

eFigure 1. Determination of retention in care: three illustrative patient examples
